# Network development in workplace health promotion – empirically based insights from a cross-company network promoting physical activity in Germany

**DOI:** 10.1186/s12889-024-19025-4

**Published:** 2024-06-10

**Authors:** Madeleine Gernert, Gabriele Fohr, Andrea Schaller

**Affiliations:** 1 Department of Neurology, Psychosomatic Medicine, and Psychiatry, Institute of Movement Therapy and Movement-Oriented Prevention and Rehabilitation, German Sport University Cologne, Cologne, Germany; 2 Institut für qualifizierende Innovationsforschung und -beratung GmbH, Bad Neuenahr-Ahrweiler, Germany; 3 Institute of Sport Science, Department of Human Sciences, University of the Bundeswehr Munich, Neubiberg, Germany

**Keywords:** Social network analysis, Workplace, Health promotion, Occupational health promotion, Physical activity, Interorganisational network, Interorganizational network, Network management

## Abstract

**Background:**

In the field of health promotion, interorganisational networks are of growing relevance. However, systematic and target-oriented network management is of utmost importance for network development. The aim of this article is to report on the development of a cross-company network promoting physical activity, and to identify necessary activities and competencies for a systematic network management.

**Methods:**

The network was systematically planned and implemented in a German technology park comprising different companies. To assess and describe the development of the network, quantitative social network analysis was conducted. To answer the question on the activities and competencies for systematic network development semi-structured interviews with participating stakeholders, and a focus group discussion with health promotion experts were conducted. The interviews were analysed deductively and inductively with the structuring content analysis method and the focus group discussion was analysed deductively by summarising key aspects of the discussion.

**Results:**

Network metrics showed that the network became larger and denser during the planning phase, and stagnated during the implementation phase. As key facilitators for network development, participation of all stakeholders, a kick-off event, and the driving role of a network manager were identified. Necessary activities of the network manager were related to structural organisation, workplace health promotion offers, and cross-sectional tasks. The results suggested that not only professional and methodological competencies, but also social and self-competencies were required by the manager.

**Conclusions:**

Our study provides initial guidance regarding the activities and required competencies of an interorganisational network manager. The results are of particular relevance for the context of workplace health promotion, since a network manager can be considered as a driving role for planning and implementing a cross-company network.

**Trial registration:**

The study is registered in the German Clinical Trials Register (DRKS00020956, 18/06/2020).

## Background

Interorganisational networks have become indispensable in the health sector [1, 2, 3]. Meanwhile, the network approach has spread from healthcare to the fields of public health and health promotion [4, 5]. However, for successful and sustainable networks, targeted network management is supposed to be an important factor for network development [6, 7].

Network research is emerging in the health sector [8, 9]. So far, research was mainly related to social networks on a micro level and their impact on individual health focussing infectious disease transmission [10], social support [11, 12], health behaviour [13, 14], and health inequalities [15]. However, on a meso level, interorganisational networks could also impact individual health [16]. Apart from various definitions [17], interorganisational networks can basically be defined as network structures between organisations [18]. The approach of interorganisational networks intends to facilitate knowledge translation and promote diffusion and sharing of information and resources between network members [19, 20]. According to literature, interorganisational networks improve quality and safety in healthcare [16, 21, 22], as well as in the public health sector [23, 24].

In the field of health promotion, interorganisational networks are of increasing interest, as it can be assumed the cooperation of relevant stakeholders might contribute to effective and sustainable primary prevention strategies [25]. Health promoting networks have already been implemented in different settings, such as local health and wellbeing services [26, 27]. Some of them addressed specific target groups, like students [28] or overweight people [29]. Interorganisational networks can also focus on particular health outcomes, e. g. chronic disease prevention [30] or physical activity promotion [31].

In Germany, interorganisational networks have become particularly interesting in the context of workplace health promotion (WHP), which is the largest setting for prevention interventions in terms of financial expenditure [32]. Since the National Prevention Strategy calls for an integrated approach across providers and sectors [25], networking and counselling are one of the preventive fields of action to address in WHP [33]. The network approach is particularly intended to facilitate access to WHP programmes for small companies, as they are less likely to provide WHP offer for their employees [34, 35]. According to the Leitfaden Prävention (Guideline Prevention), cross-company networks comprise at least two companies and a social health insurer [33]. The network partners (e. g. including company representatives, regional actors) agree on certain goals, tasks and rules of cooperation in the context of WHP [33]. As a result, cross-company networks can be classified as interorganisational networks.

Since interorganisational networks are usually difficult to develop [36], management is considered important [37]. In the field of public affairs, network structure, management strategies, and outcomes of collaboration have been qualitatively identified as interrelated factors in interorganisational networks [38]. In public education, it was possible to quantitatively show an effect of network management on an education program, since network managers’ engagement in interactions improved program performance [6]. According to literature in the context of government activities, network management can be defined as “strategies aimed at facilitating and guiding the interactions and/ or changing the features of the network with the intent to further the collaboration within the network processes” [7]. In this respect, selecting network partners, task allocation, defining resources and responsibilities as well as the coordination of collaboration, and the evaluation of interorganisational relations are considered of utmost importance for interorganisational network management [39]. Regarding the responsibility for these activities, literature on interorganisational networks in different contexts proposes three types of network management [17]. First, shared governance networks are collectively managed by the network members themselves which mostly occurs in networks in business or private industry [17]. Second, lead organisation governance occurs when a network member leads the network [17]. Third, network administration organisation (NAO) government is characterised by an external leading organisation or individual which is not part of the network itself, but specially created to manage the network [17]. According to Provan and Kenis [40], each form of network governance has its own strengths and weaknesses, and the choice of which form suits best, depends on “four key structural and relational contingencies” (p. 237), namely trust, size, goal consensus, and the nature of the task. The form of NAO government was found to occur most in the context of health and human services and is considered to enhance interactions between the public and private sector [17].

Despite the strong need for management activities in health networks [20], knowledge about the structure and functioning of these networks remains limited [41]. However, it can be assumed that an appropriate network management facilitates targeted network development aligned with the respective network goal. Therefore, it can be assumed that cross-company networks do also need management to sustainably achieve their goals, such as health promotion [18, 20, 42].

Thus, the present study aims at contributing to the knowledge on how to develop and manage a health promoting cross-company network on the organisational level. The related research questions are:

- How has a health-promoting cross-company network developed on the organisational level?
- What activities and competencies are required to manage a health-promoting cross-company network?

## Methods

**Fig. 1:**
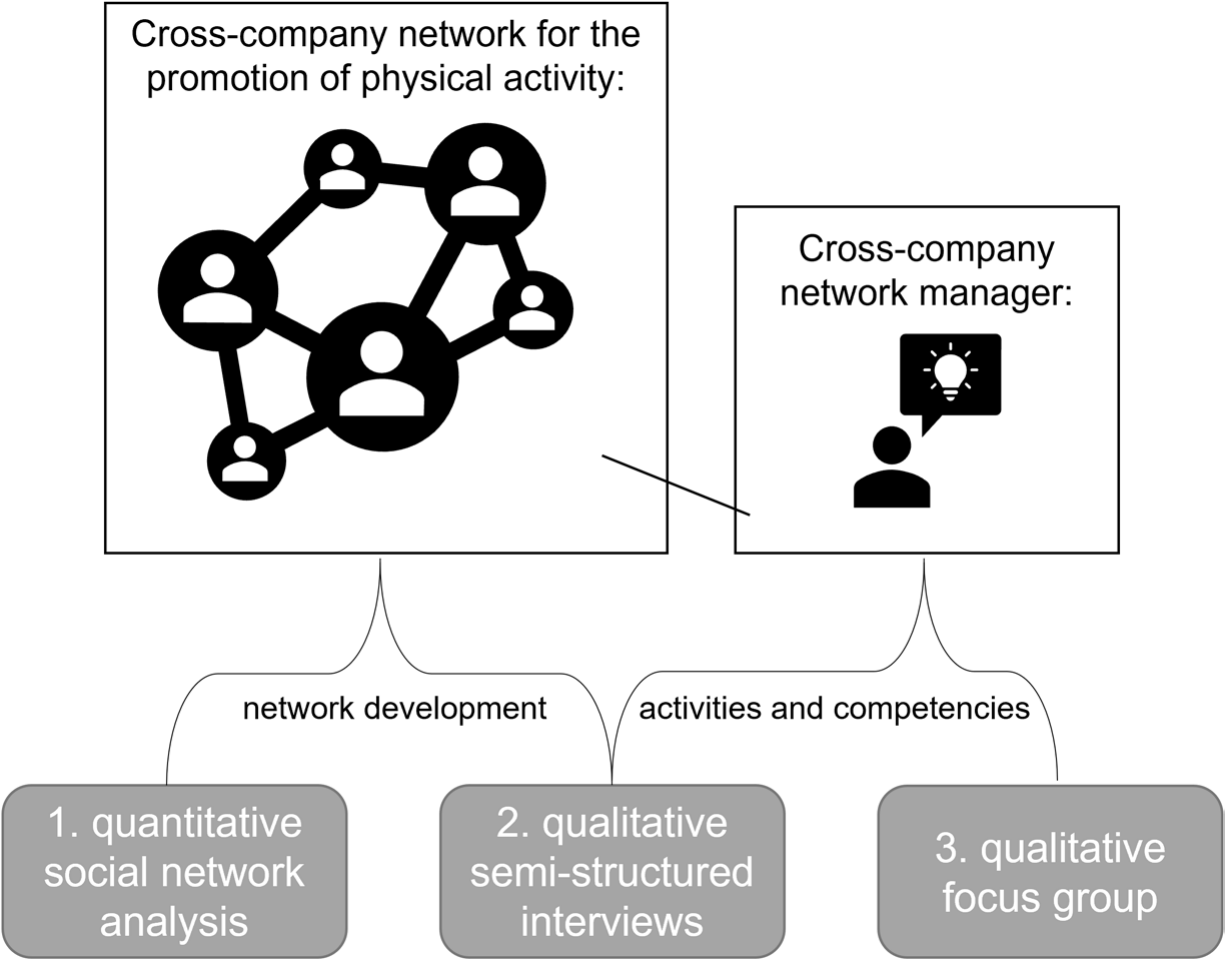
The mixed-methods study design includes quantitative social network analysis and qualitative semi-structured interviews to describe network development. Qualitative approaches examine the cross-company network manager’s activities and required competencies

The present mixed-methods study combines a quantitative social network analysis (SNA), with qualitative semi-structured interviews and a focus group (Fig. 1). The evaluations were conducted within the KomRueBer project which took place in a technology park in Germany from July 2019 to May 2022. The aim of the KomRueBer project was to develop, implement, and evaluate a cross-company network for the promotion of physical activity, as an example for health promotion. A steering group, consisting of company representatives, exercise providers, and network partners, participatively concepted a multicomponent intervention and steered its implementation. Company representatives were employees or leaders of the participating companies (for example of a technological enterprise). Exercise providers offered the WHP interventions for the cross-company network (for example a gym or a health coach). Network partners included representatives from society/politics (for example health insurance funds, the pension fund, or municipal administration), and from public or economy (for example business development agencies, media representatives). Thereby, a so-called cross-company network manager was responsible for the coordination of steering group events and interventions within the network. The cross-company network manager was employed by a WHP provider not belonging to the technology park. Within the project, participation in the cross-company network was not associated with any costs for the companies. A study protocol has been published [43], the trial was registered in the German Clinical Trials Register (18/06/2020; DRKS00020956), and ethics approvals were received (German Sport University Cologne; reference numbers 120/2019 and 068/2020).

### Social network analysis

To answer research question 1 (How has a health-promoting cross-company network developed on the organisational level?), a quantitative social network analysis was conducted to examine the development of the KomRueBer cross-company network for the promotion of physical activity on the organisational level. Detailed methodology of the approach applied and first results have been published elsewhere [44, 45].

#### Setting and data collection

Overall, seven network events from August 2018 to May 2022 were considered in the present SNA analysis: T0 (08/2018): letter of intent, before project start; T1 (07–09/2019): on-site information and consultation; T2 (07/2019): steering group I; T3 (01/2020): steering group II (finalisation of the multi-component intervention); T4 (10/2020): digital steering group III; T5 (06/2021): digital steering group IV; T6 (05/2022): digital steering group V. Except for T0, which was prior to the project start, all network events were planned and delivered by the manager of the cross-company network. The project was divided into a planning phase (T0 to T3) and an implementation phase (T4 to T6).

#### Data analysis

The following global network measures were calculated: network size (number of nodes, number of edges), degree measures (average degree, average weighted degree), distance measures (network diameter, average path length), density and clustering measures (network density, total number of triads, average clustering coefficient) [44, 46, 47].

### Semi-structured interviews

To answer research question 2 (What activities and competencies are required to manage a health-promoting cross-company network?) semi-structured interviews and a focus group were conducted. Semi-structured interviews explored facilitators and barriers in the development of the KomRueBer cross-company network for the promotion of physical activity. Additionally, the activities of a cross-company network health promotion manager were discussed from the participants’ point of view. This qualitative approach is reported based on the consolidated criteria for reporting qualitative research (COREQ) [48].

#### Participants

All 18 stakeholders involved in the KomRueber project were invited to participate in a semi-structured interview by the KomRueBer network manager via email or telephone. Four of the invitees did not answer to the request. 14 stakeholders (four male, ten female, mean age 47 ± 8) provided informed consent in voluntary participation in the interviews. The participants were assigned to their predominant role in the network which was either network partner (*n* = 8), or exercise provider (*n* = 4), or company representative (*n* = 2).

#### Setting and data collection

The interviews were held in German language, in August and September 2021. Due to the COVID-19 pandemic, the interviews were conducted via online video conferences. The semi-structured interview guide was internally pilot-tested and addressed two main topics. Firstly, the cross-sectional network for the promotion of physical activity was discussed. Afterwards, the implemented communication strategy during the KomRueBer project was addressed. The results concerning the implemented communication strategy have been published elsewhere [49]. The present paper focuses on the first part of the interviews, discussing the experienced facilitators and barriers during the implementation and realisation of the cross-company network for the promotion of physical activity, and the roles of different stakeholders within the network, especially the role of the network manager.

The interviews were conducted by a female Ph.D. candidate, scientist and WHP practitioner who also was the KomRueBer network manager. She was trained in qualitative research and had no relationship with the interviewees apart from the KomRueBer project.

All interviews were anonymised by a code and digitally recorded. Data collection was completed by handwritten field notes and demographic data which was gathered after the end of the interview.

#### Data analysis

The complete interview records lasted from 35 to 70 min (mean: 50 ± 11 min). The first part of the interview, which is subject of the following analysis, lasted from 15 to 40 min (mean: 25 ± 7 min). A professional typist transcribed the records according to Dresing and Pehl [50]. Afterwards, the transcripts were analysed using the structuring content analysis method [51, 52]. The structuring content analysis method is comparable to the framework method for the analysis of qualitative data [53]. Data analysis was carried out by two researchers. Firstly, thematic main categories were developed deductively from the interview guide and inductively from the transcript. Then the material was coded according to the main categories. Afterwards, the research team inductively derived subcategories for each main category. The researchers discussed and adjusted all main and subcategories before they analysed and coded the dataset accordingly, using MAXQDA Standard 2020 software (VERBI GmbH Berlin). Finally, the material of each category was summarised. A professional translator translated particularly meaningful quotes into English for the publication of results.

### Focus group

According to research question 2 (What activities and competencies are required to manage a health-promoting cross-company network?), a focus group was conducted to describe the activities of a cross-company network health promotion manager from an experts’ point of view and to identify the required competencies. The method is reported on the basis of the consolidated criteria for reporting qualitative research (COREQ) [48].

#### Participants

The focus group consisted of the KomRueBer project staff (one male, five female) and therefore was a heterogenous, natural group. The group included two representatives from the German Sport University Cologne (GSU), one person from the Institute for Occupational Health Promotion (IOHP), and two representatives from the Institut für qualifizierende Innovationsforschung und -beratung GmbH (IQIB). The network manager, who also participated in the focus group, was employed both at the GSU and the IOHP. Each member of the project staff participated in the focus group since the results were meant to contribute to a sub-goal of the research project.

#### Setting and data collection

A project member set the appointment for the focus group which took place on 15.12.2020 and lasted two hours. Due to the COVID-19 pandemic, the focus group was held with an online meeting tool (Cisco Webex Meetings, Cisco Systems, Inc., Milpitas, California, USA) and supported by an online whiteboard (mural board, MURAL, San Francisco California, USA). Thus, there was the opportunity to share contents and to comment on them on-screen.

The two-parted focus group discussion was conducted in German language and followed a structured guide including a general orientation phase and phases of introduction, elaboration, and consolidation for each topic. In the first part of the discussion, a profile of the cross-company network manager’s activities was elaborated. In the second part, the demands on this profile and the resulting necessary competencies of a network manager were identified. Thereby, the discussion on competencies was based on Kauffeld’s four competency facets: professional, methodological, social, and self-competency [54]. The competencies were also discussed against the background of the COVID-19 pandemic and the resulting rise of digitalisation of WHP interventions. Prior to application, peers tested the comprehensibility of the questions, and the project leader approved the question guide.

The focus group was held by a female master student as a part of her master’s thesis. Prior to the master’s thesis, she had no relationship to the KomRueBer project and project staff.

The online meeting was audio- and video-recorded. Additionally, the moderator took field notes on the mural board during the discussion, and saved them for following analysis.

#### Data analysis

The record of the focus group discussion lasted 128 min. After the recording, an abridged transcript was made [55]. The field notes on the mural board were checked for accuracy and completeness using the transcript and audio record. After that, the material was analysed deductively by summarising key aspects of the discussion [56], which is related to Lamnek [57] and Mayring [58]. Accordingly, categories were formed that subsumed central discussion aspects. A discussion aspect is considered central if it appears repeatedly in the group discussion and is discussed by the participants in a certain depth and breadth [56]. Firstly, regarding the cross-company network manager’s demands and required competencies, the data material was categorised on the basis of Kauffeld’s four competency facets [54]. Beyond these results, inductive categories were developed on the basis of the transcript. Secondly, the material of each identified category was reduced to a central statement. Thirdly, concise quotations were selected from the audio record to illustrate central discussion aspects, and translated into English by a professional translator.

## Results

### Social network analysis

At T0, the KomRueBer network consisted of a total of 9 actors (4 exercise providers, 5 network partners, 2 companies) and expanded to 23 actors at T6 (8 exercise providers, 8 network partners, 9 companies). From the 9 participating companies, one company also was an exercise provider, and one company also was a network partner. Two participating companies had up to 9 employees, three had 10 to 49 employees, three had 50 to 249 employees, and one company had more than 250 employees. The participating companies mainly belonged to the service sector and manufacturing industry. Table 1 displays the visualisation of network development by two-mode networks showing the participation of the actors (nodes) at an event and one-mode networks showing the connection of organisations by their common participation at events. Network metrics of the cross-company network during the project course refer to the one-mode network.

**Table 1:** Visualisation and network metrics of the cross-company network during the project course [45]

	**T0**	**T0-T1**	**T0-T2**	**T0-T3**	**T0-T4**	**T0-T5**	**T0-T6**
**Two-mode network: participation of the actors (nodes) at an event**							
Visualisation							
**One-mode network (projection of the events onto the edges): connection of organisations by their common participation at events**							
Visualisation							
Number of nodes	9	17	20	22	23	23	23
Number of edges	36	72	106	148	153	153	155
Average degree	8	8.471	10.6	13.455	13.304	13.304	13.478
Average weighted degree	8	8.471	12.7	17.545	18.087	19.391	19.913
Network diameter	1	2	2	2	2	2	2
Average path length	1.0	1.4	1.442	1.359	1.395	1.395	1.378
Network density	1.000	0.529	0.558	0.641	0.605	0.605	0.613

*T0* Letter of intent, *T1 *On-site information and consultation, *T2 *Steering group I, *T3 *Steering group II, *T4 *Digital steering group III, *T5 *Digital steering group IV, *T6 *Digital steering group V, *red *Organisations, *green *Events, *node size *Degree Centrality

The visualisation of the two-mode network showed an enlargement of the network, as the number of participants (nodes) and connections (edges) increased over time. Additionally, the average number of connections per participant also increased. In the T6 network, 15 organisations had participated in two or more network events.

In the graphics of the one-mode network is noticeable that from T4, 12 organisations formed a core network as they were connected more densely. The subgroup is composed of different stakeholders, with network partners from society/ politics and economy being represented as particularly central actors.

The metric measures of the one-mode network confirmed the previously observed network expansion and densification. In summary, the network metrics quantified the increase of the network from initially 9 to 23 actors (nodes), and from 36 to 155 connections (edges). However, the network metrics hardly changed after T4. The average degree (average number of connections of a single actor to others) increased from 8 (T0) to 13 at T3 (steering group II), then decreased slightly and stagnated (T3-T5), but increased again at the digital closing event (T6). Meanwhile, the edges of organisations that met more frequently were strengthening, which was expressed by a continuously increasing average weighted degree. The closeness of actors in the network was supported by the maximum network diameter of 2 and an average path length (average distance between all pairs of nodes) between 1.359 and 1.442. Network density refers to the number of existing edges between actors compared to the maximum of possible edges. At T0, all actors met for the first time. As a result, each actor shared a connection with every other actor (network density = 1). At T1, network density decreased, as not all participants from T0 also attended the event at T1 (0.529). Afterwards, network density increased over the course (T2: 0.558; T3: 0.641), and remained stable at a value round 0.6 (T6: 0.613). Accordingly, around 60% of the possible connections in the network developed during the project. Simultaneously, the density and clustering measures showed a steady but weakening increase in the total number of triangles (T0: 84; T6: 549). In contrast, the clustering coefficient decreased from 0.969 (T1) to 0.842 (T6), reflecting a continuous enlargement, except for the stagnation between T4 and T5.

### Semi-structured interviews

Overall, five main categories about facilitators and barriers in the cross-company network for the promotion of physical activity were identified: *individual participation in WHP*, *organisational participation in the network*, *contextual factors*, *network management*, and *suggestions for improvement*. For each main category, subcategories and characteristics were built inductively. Table 2 shows an overview of the main categories, the related subcategories, and subordinate characteristics.

**Table 2:** Overview of the main categories and subcategories

Main categories	Subcategories	Characteristics
***individual participation in WHP***	themes of the WHP offers	• facilitator • barrier
	format of the WHP offers	
	access options to WHP offers	
	visibility of WHP offers	
***organisational participation in the network***	value of WHP in the participating organisation	• facilitator • barrier
	internal resources for WHP	
	reasons for participation in the cross-company network	
	internal structure of the organisation	
***contextual factors***	infrastructure	• facilitator • barrier
	management of the technology park	
	seasonal impact	
	COVID-19 pandemic	• impact on individuals • impact on organisations
***network management***	developing the cross-company network	• facilitator • barrier
	project management	
	conception, organisation, and quality assurance of WHP offers	
	network fostering	
	public relations of the network	
	required competencies of the network manager	• professional competencies • personal competencies
***suggestions for improvement***	increasing the number of WHP participants	
	enhancing the networking of organisations	
	project management	
	continuation of the project	

#### Individual participation in WHP

The main category *individual participation in WHP* comprised the determinants of participation in WHP in the cross-company network at the individual level of the targeted employees. Four subcategories were identified: *themes*, *format*, *access options* and *visibility of WHP offers*.

Regarding the *themes* it was pointed out, that the needs and potential barriers of the target group, e. g. regarding topics, group composition, or schedule, need to be taken into account. On the one hand, it was suggested to offer a wide range of interesting activities. On the other hand, a too broad distribution of themes could lead to less participation in each single offer.

Regarding the *format,* several facilitating characteristics of online courses were pointed out. They were described as low-threshold and individualized while providing an offer for many employees at the same time. Therefore, online offers were considered a good alternative to face-to-face courses. Nevertheless, WHP providers explained that online supervision was more difficult due to reduced contact to participants and some statements considered face-to-face formats superior to online formats.

*“Well, at the end of the day, the interaction with each other, in sports and in the workshops, is what adds value, I guess.” (HE911C, 19)*

For future WHP offers, many interviewees advocated offering hybrid formats, if possible.

*Access options* dealt with the accessibility of WHP offers. Low-threshold WHP offers were characterised by taking place at the workplace and during working hours or being compatible with working hours. As a main barrier for the target group’s access to WHP offers, the interviewees reported that many employees did not receive newsletters about the WHP offers due to the absence of a kick-off event.

*“In my opinion, the kick-off event would have been a really big key to success. Because those super-motivating newsletters you created would have reached the ones they were supposed to reach. Being as motivating as they are, they also would have convinced them to participate.” (ET510C, 23)*

Moreover, employees might have experienced barriers trying to integrate WHP offers into their daily working routine or asking their supervisor for agreement. Some statements proposed that trial offers could be a useful tool to reduce possible barriers to participation.

*Visibility of WHP offers* dealt with the perception of WHP offers taking place. To increase the employees’ interest in WHP offers, the interviewees emphasized that offers should be promoted repeatedly, for example by trial offers, events, promotion days, newsletters, the website, and the offers themselves. However, in the present project, the respondents assumed that the target group did not receive enough information on WHP.

#### Organisational participation in the network

The main category *organisational participation in the network* contained characteristics, structures, and motives of the organisations that are part of the cross-company network. Four subcategories were identified.

Regarding the *value of WHP in the participating organisation*, the interviewees assumed that organisations were more likely to participate in the cross-company network if the leadership was already interested in physical activity and health promotion. While strong company leaders’ support could increase financial resources and facilitate employees’ participation in WHP, low value of WHP in a participating organisation could inhibit the whole network.

*“And I think it doesn’t make sense to somehow drag along a company that is involved without a commitment. Therefore, I think it certainly was the right way to say: I’ll go with those who say they want to do this.” (OA83C, 35)*

The most frequently named factor influencing *internal resources for WHP* is the company size, since small companies do not have sufficient resources for their own WHP programmes. Nevertheless, joining and supporting the cross-company network also required companies to invest both time and budget, which might be a barrier, especially for small companies, *“because you have to do that on top of your regular job” (HE911C, 15)*.

*Reasons for participation in the cross-company network* summarised the organisations’ motives and concerns regarding participation. For company representatives, the focus was on networking with other companies, not only in the context of physical activity. WHP providers mainly used the network to advertise their own services, as for *them “it is important to use all cost-free channels reaching local people” (NE81C, 89).* The network partners were interested in gaining knowledge on network development for future cross-company networks and considered networking and health promotion as relevant topics. The interviewees agreed, that network partners should not participate solely for economic reasons.

In the subcategory *internal structure of the organisation*, multipliers were highlighted as having a key role in the internal implementation of WHP by ensuring the dissemination of information to the target group. However, especially in small companies, it was hard to find a suitable multiplier. Moreover, the multiplier approach led to a bottleneck situation and it was unclear how much of the intended information really reached the target group.

*“I mean, there is always the question: To what extent is the information forwarded and received? Of course, that is a difficulty. (…). Of course, we are sending this information to someone in the company. And then you are somewhat exposed. Is it forwarded or not? And how is it carried forward? This is a real, classical bottleneck.“ (OA83C, 37)*

#### Contextual factors

The main category *contextual factors* included external factors influencing the cross-company network. Four subcategories were identified.

It was considered beneficial to use and develop the surrounding *infrastructure* that already existed, e. g. sharing facilities like showers and changing rooms. Especially in smaller companies, facilities were limited.

The *management of the technology park* was described ambivalently in the interviews. On the one hand, the property management had a strong interest in the success of the network resulting in a positive impact for the technology park. On the other hand, WHP seemed to have a low priority for the property management and there were restrictions that inhibited the network.

*“And that is where the difficulty lies, that there is not that much willingness.” (RS19C, 17)*

Regarding the *seasonal impact* on the cross-company network, the respondents advised that WHP offers should suit the season. For example, more indoor offers should be scheduled in the cold seasons. Despite some interviewees describing the seasonal influences as rather small, others reported that WHP offers get the best participation rates at the turn of the year, in spring and autumn, and after holidays. The summer holidays were mentioned as the most inconvenient time due to low demand and resulting participation in WHP.

The central context factor *COVID-19 pandemic* showed a significant impact on the cross-company network at the individual and organisational level. On the individual level, the pandemic caused the cancellation of a kick-off event and accessibility to the workforce was limited due to many employees working from home.

*“A kick-off event was planned. Due to COVID-19, it was cancelled quite spontaneously. And thus, from my point of view, the whole project took a completely different course than we had expected. Because right from the start I thought that – even though there was a lot of commitment from all stakeholders – the target group was very difficult to reach.“ (TN114C, 11)*

As a result, WHP offers had to take place online (view *format of WHP offers*). Despite vaccinations, the fear of an infection seemed to be a continuing barrier to participation when face-to-face offers were reintroduced. On the organisational level, some WHP providers were not able to offer new courses to the network because they were trying to compensate their loss of clients and coaches due to the previous restrictions. Regarding the companies, the respondents had the feeling that the systematic integration of WHP fell short and due to lasting changes to the work environment, it *“is not yet known how it will proceed “ (GA06C, 13).*

#### Network management

The main category *network management* involved tasks related to the management of the cross-company network and requirements for the responsible network manager. Six subcategories were identified.

For the task of *developing the cross-company network* interviewees recommended to personally contact the companies, and to assure a high quality of WHP offers. According to the interviews, a kick-off event with all participating organisations could be a comfortable way to meet each other. In addition, all stakeholders should be engaged in the network development to increase feasibility, acceptance, and usage of the WHP offers. As barriers for network development, the interviewees mentioned finding appropriate contact in the participating organisations, and keeping the network at a manageable size, *„because it also becomes more difficult in terms of communication. So, then you should also take a cut at some point “ (KK65C, 21).*

Regarding *project management*, the network manager steered the project with a goal-oriented approach and served as contact for all stakeholders in the cross-company network. In this regard, the interviewees appreciated a participative approach with a friendly working atmosphere. The network manager was also responsible for ensuring the funding and evaluation of WHP offers. A clear challenge was that the network management was a resource-intensive task which could not be provided by most participating organisations. Therefore, the interviewees assumed that without an external network manager, the network was unlikely to continue.

*“‘cause somebody has to take responsibility for this again, (...), yes, (laughter) because without active leadership, it would just disappear again and that would be a pity.” (DR313C, 57)*

Regarding the WHP offers, the interviews revealed the tasks *conception, organisation, and quality assurance*. During the conception of WHP offers, the needs and interests of employees, structural and financial conditions, and the capacities of WHP providers should be considered. For quality assurance, some interviewees recommended certified WHP providers and regular evaluations. However, the restriction to certified WHP providers could be a major barrier to network development.

*Network fostering*, defined as measures to consolidate the network through communication and information, was described as a continuous task of the network manager. Information dissemination via motivating newsletters, a website, and especially personal meetings were considered positive. Meetings facilitated exchanges between companies and collaboration of all stakeholders. An inevitable barrier for network fostering was that meetings could only be conducted online and that newsletters and the website, while informative, did not promote networking between companies. As a result, the participants reported only few contacts with other stakeholders.

*“In my opinion, it simply wasn’t possible to develop a really stable network. There weren’t enough meetings for that, I think. And they were only digital meetings.“ (OA83C, 63)*

Regarding *public relations (PR)* of the network, the stakeholders proposed to present the network to potential future network partners. The interviewees proposed to use the website, events, visible WHP offers, and personal approaches to spread positive examples of WHP implementation. Posters were described as too passive for the purpose of PR.

The interviews outlined *required competencies of the network manager*. As professional competencies, expertise in the fields of sport science, WHP, and the Leitfaden Prävention were named, as the manager is working at this interface. Additionally, various personal competencies were considered necessary for the development and management of a cross-company network: extroversion, openness, sociability, friendliness, empathy to accompany and motivate network partners, engagement, perseverance, and a structured working attitude.

#### Suggestions for improvement

The main category *suggestions for improvement* included proposed improvements and desirable modifications for the cross-company network. Four subcategories were identified.

For *increasing the number of WHP participants*, the interviewees regarded a kick-off event (see above) as key to reach the target group, and to collect contact information for newsletters. Additional communication tools like printed t-shirts or company visits were seen as ways to raise the employees’ attention.

In order to *enhance the networking of organisations*, the interviewees argued that personal contacts were more binding than written information. In addition, positive experiences of already participating organisations or bottom-up approaches of the employees were seen as useful approaches to attract the organisations’ decision makers.

*“The direct contact, and then perhaps also to show some positive examples to the companies, and to tell them who has already participated, or is participating, and how the planned project looks like.” (RS19C, 15)*

At least the respondents wished for more opportunities for networking between companies, for example sharing existing WHP offers.

Regarding *project management*, the respondents underlined the important role of the network manager and wished for more regular meetings of the network partners.

*“Of course, I would have like to hold more face-to-face meetings, so that I could talk to people in person.” (KK65C, 23)*

In order to expand the network, many interviewees proposed personal meetings with company leaders and the involvement of health insurers.

Most of the participants desired a *continuation of the project*, with network development provided by a network manager. However, some participants expected the network to end due to leadership vacancy.

*“Yes, at the end of the day, the central role/ you would actually need a continuing/ someone who maybe continues, I mean project management. And I don’t see who will take on that role.” (NR412C, 45)*

Physical activity promoting WHP offers could be complemented by other themes (e. g. diet and weight management, stress management).

### Focus group

#### Activities of the cross-company network manager

**Fig. 2:**
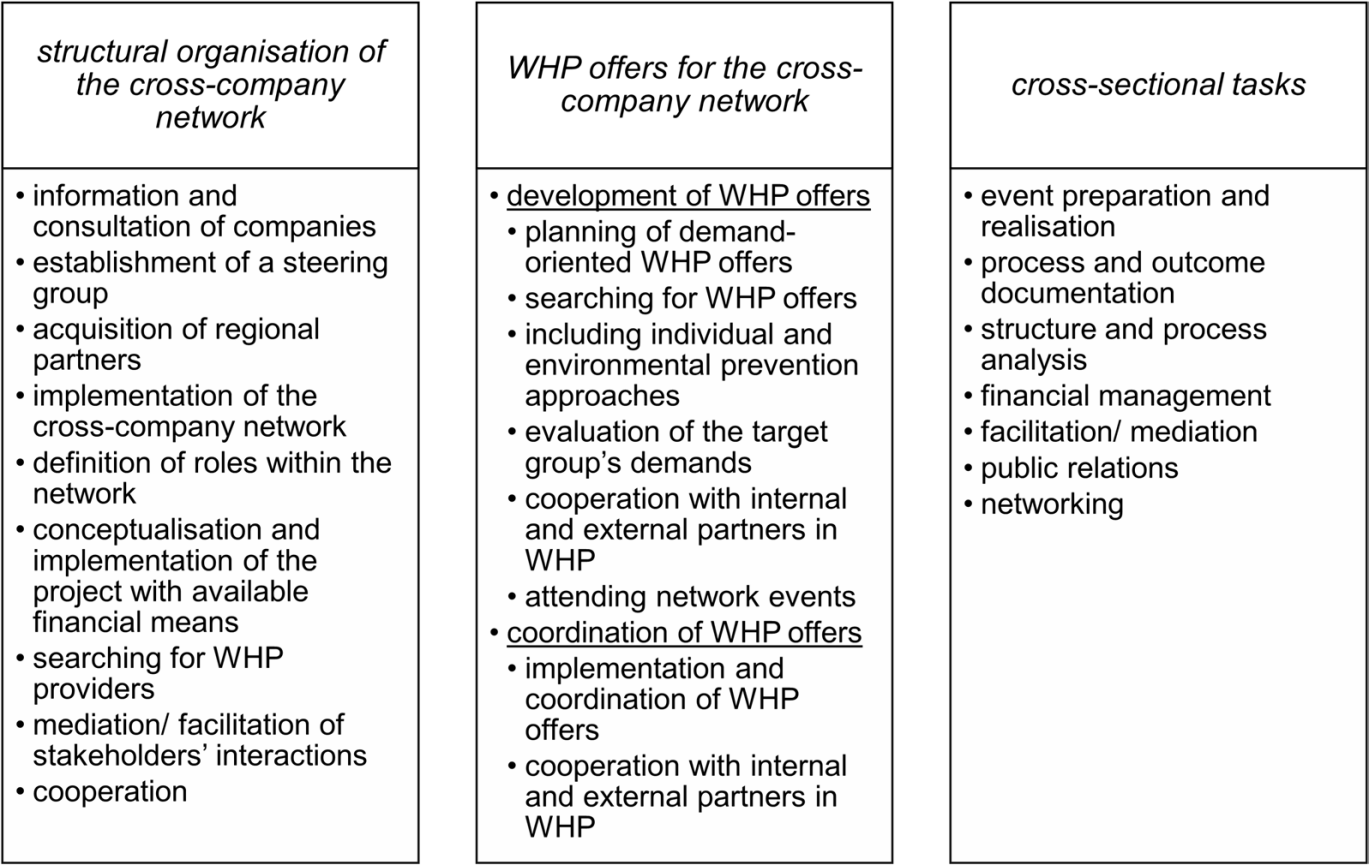
Activities of the cross-sectional network manager

The activities of the cross-company network manager mentioned by the focus group were structured and summarised to the inductive categories *‘structural organisation of the cross-company network*, *‘WHP offers for the cross-company network*, and *‘cross-sectional tasks’* (Fig. 2).

Activities in the category *structural organisation of the cross-company network* predominantly referred to engaging different stakeholders in the network and facilitating interactions.

*“Acquiring all the regional partners. First of all, I actually have to find them. That’s already very time-consuming.” (E6, 130)*

Regarding *WHP offers for the context of the cross-company network*, the categories *‘development of WHP offers’* and *‘coordination of WHP offers’* arose. While *development of WHP offers* mainly comprised the search for and choice of appropriate WHP offers, *coordination of WHP offers* referred to the implementation and coordination of WHP offers with internal and external WHP partners.

*“Also, for the demands... i.e., the analysis of the current situation, for the demand-oriented offers.” (E4, 139)*

As *cross-sectional tasks*, activities were named in the context of events, administration, and communication.

*“Yes, just like that, conducting the steering group meetings, informative meetings, just as you say E5.” (E2, 149)*

#### Required competencies of a cross-company network manager

**Fig. 3:**
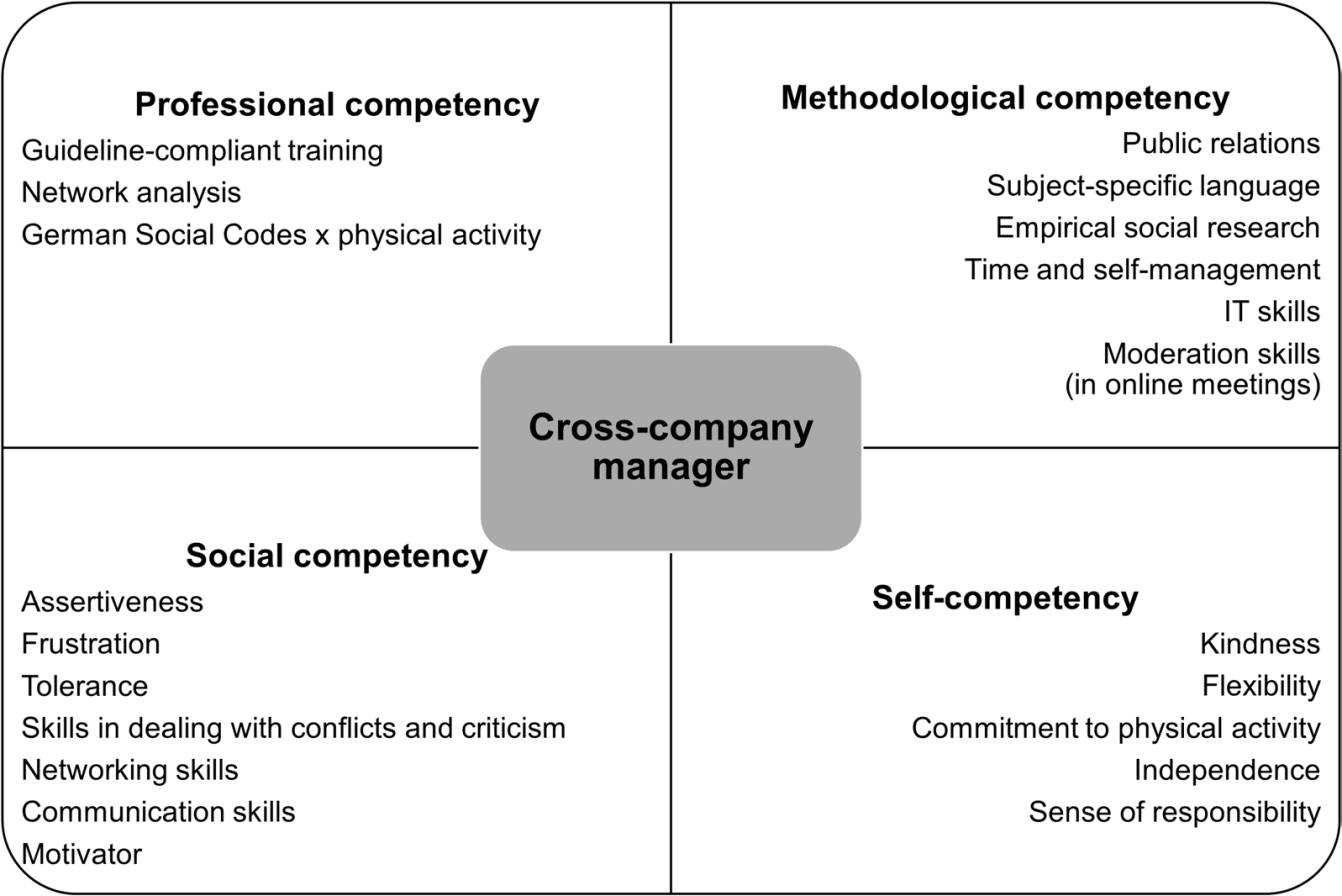
Required competencies of a cross-company network manager structured on the basis of Kauffeld’s four competency facets

The required competencies to manage a cross-company network named by the focus group were structured according to Kauffeld’s four competency facets (Fig. 3) [54].

As *professional competencies*, the focus group named skills in network analysis and knowledge of the legal conditions of physical activity promotion in Germany. Additionally, the educational training of the cross-company network manager should be compliant to the German Leitfaden Prävention. *Methodological competencies* were related to public relations, subject-specific language, methods of empirical social research, time and self-management, and IT and moderation skills for online meetings. As *social competencies*, the focus group identified assertiveness, frustration tolerance, skills in dealing with conflicts and criticism, in networking, and communication. In addition, the cross-company network manager was called a *“mega-motivator” (E6, 245).* Finally, as *self-competencies*, the experts referred to kindness, flexibility, commitment to physical activity, independence, and a sense of responsibility.

As the five most important competencies of the cross-company network manager, the focus group exposed (1) management competency, (2) specialist knowledge (physical activity, legal conditions), (3) organisational talent, (4) the ability to enthuse, and (5) communication competency.

## Discussion

SNA results showed a densification and continuous enlargement of the network in the planning phase with subsequent stagnation in the implementation phase. Key facilitators for the network development were a kick-off event and the participation of all stakeholders. Network management activities were related to the context of structural organisation, WHP offers, and cross-sectional tasks. This was not only related to professional and methodological competencies, but also to social and self-competencies.

Regarding the network development, studies underline the importance of network engaging events such as kick-off events [20] and regular meetings to maintain networks [41]. Since the COVID-19 pandemic in 2020 led to a complete lockdown in Germany with strict contact restrictions for several weeks, this was no longer possible. Hence, the negative consequences of the pandemic on network development and stabilization are quite obvious. This is also reflected in the results of the semi-structured interviews, where stakeholders reported difficulties in establishing new contacts and reaching the target group. For example, the pandemic caused the cancellation of a kick-off event at the transition from the planning to the implementation phase (between T3 and T4). Additionally, stakeholder meetings and WHP offers had to be reorganized at short notice to online offers. However, the quantitative SNA metrics of the established network (since T4) showed, that the density of the KomRueBer network was even higher than in comparable networks [28, 41].

Our results emphasize the relevance of interorganisational networking in (workplace) health promotion, as a way to increase health promotion capacity of small companies. In the context of WHP, health promotion capacity has been proposed as a key success factor, implying “health promotion willingness” and “health promotion management” [59]. Thereby, “health promotion willingness” is defined as the willingness of a company to implement WHP on a permanent basis [59]. In line with this, our results showed that organisational commitment and engagement, as well as a high value of WHP in the participating organisations were important for both, their individual internal development of WHP, and for the cross-company network development. Regarding “health promotion management”, referring to the extent to which WHP is being put into practice systematically [59], the stakeholders emphasized the importance of the networking approach in the interviews. They reported, that small companies often do not have enough resources to establish their own WHP offers, which is in line with a previous investigation [35]. Company size is a major factor influencing the existence of WHP offers, as compared to small companies (< 49 employees), medium-sized companies (50–249 employees) are more than twice as likely to offer WHP and large companies (> 250 employees) are more than five times as likely [60]. Small companies with high “health promotion willingness” might regard the network approach as a way to facilitate the access to WHP offers, if a network manager dealt with “health promotion management”. Indeed, since the German Leitfaden Prävention promoted networking as a possible way to engage small companies in WHP [33], a higher proportion of these companies could be observed in cross-company networks than in WHP offering companies in general [32]. This observation suggests that the WHP network approach should be encouraged in the future. Thereby, appropriate network management should be supported.

The role of the network manager was a central part of the KomRueBer project. The results of the semi-structured stakeholder interviews as well as the expert focus group, pointed out the driving role of the external cross-company manager. Our results on the importance of network management activities for network development and success are in line with previous studies on health- and physical activity-promoting networks [42, 61, 62]. In the KomRueBer project, the network manager represented the network administration organisation (NAO) which was a good choice of governance form according to the qualitative interviews in our study. Although the appropriate form of network governance generally depends on several structural characteristics of the network, in terms of network development, NAO governance and lead organisation governance are considered more effective than shared-governance networks [40]. Moreover, in the context of WHP, the involvement of an independent consultant seems to be fundamental for improving employees’ health outcomes [63], which is an argument against choosing a network member for lead organisation governance. Additionally, it seems to be important to explicitly employ a network manager [20]. This was also evident in our interviews, as participating organisations refused this role due to a lack of time resources and the fact that they do not see themselves responsible for it. The network manager’s activities strongly overlap with generally working in WHP [64], as they can be subsumed under network development and fostering, organisation of WHP offers, and public relations. Regarding network development, the network manager should consider several facilitators and barriers that our study indicated. Firstly, on the individual level of the target group, employees’ needs and opportunities to participate in WHP offers should be respected. Secondly, on the organisational level, the network manager should ensure that the participating organisations’ motives match, as common aims are essential for interorganisational networks [20]. Furthermore, the manager should act proactively, initiate contacts, maintain motivation, and engage network partners [20, 65]. Thirdly, the network manager constantly has to keep in mind contextual factors influencing network development, such as the surrounding infrastructure and power structures. Facing all these activities and conditions, NAO governance requires a high level of professional and methodological competencies [18, 40]. Moreover, social competencies seem to be particularly important, as the interviewed stakeholders predominantly referred to those and it has already been shown that working in the field of health promotion does require social and self-competencies [64]. Overall, since network development is considered an ongoing and challenging process [20], perseverance and motivational competencies might be the most important ones.

## Limitations

The present study underlies some limitations. First of all, the study process was severely affected by the outbreak of the COVID-19 pandemic (lockdown in Germany in March 2020) during the transition from the planning to the implementation phase. The implementation phase could not be realized as planned. Secondly, the quantitative SNA did not include the network manager as an actor. This was due to the focus on the entire interorganisational network instead of the participating organisations themselves. However, we integrated the different levels of the entire network and participating stakeholders in our evaluation [66]. We also included quantitative and qualitative methods to a mixed methods approach to reach a comprehensive understanding of the health promoting network [67, 68, 69]. Additionally, the longitudinal design of the SNA can be considered a strength of our study because application is limited. According to a review, social network analysis generally is an appropriate method to evaluate the development of health promotion-focused networks [70]. Nevertheless, the interpretation of longitudinal SNA metrics still leaves the question of how ideal network metrics might look like which would be essential information for targeted network development [71]. Thirdly, we did not evaluate effects of the cross-company network on the physical activity behaviour of the target group. Besides common challenges of such evaluations, such as short periods of evaluations and the difficulty of measuring the individual exposure to interventions [2], we received only limited data from the target group.

Lastly, the fact that the network manager conducted the semi-structured interviews herself, and that she participated in the focus group might have caused a bias. Her involvement could have influenced the interviewees with the result that they held back with regard to critical remarks or potential for improvement of the network. However, one positive aspect to emphasise is that the subsequent data analyses were carried out by two independent researchers excluding the network manager.

## Conclusion

According to our results, the cross-company network manager had a central role in planning and implementing the cross-company network for the promotion of physical activity. Although in the present network the focus was on physical activity promotion, we assume that the results can also be applied in other fields of health promotion. Apparently, interorganisational health promoting networks need to be actively managed. As the participating organisations have stated that they are not able to handle this role, a dedicated manager is needed. Ideally, an external person would be employed as network manager. However, in this case, questions arise about funding, the employing organisation, and qualification. One solution could be a revision of the Leitfaden Prävention, explicitly outlining and describing the role of network managers as fundable expenditure for health insurers. Concerning the tasks and required competencies of a cross-company network manager, our study provides initial guidance. Regarding the effects of network management on concrete network outcomes and the achievement of network goals, more mixed-methods research is needed in the context of cross-company networks for health promotion.

## Data Availability

The data presented in this study are available from the corresponding author upon reasonable request.

## Abbreviations

COREQ: Consolidated criteria for reporting qualitative research
GSU: German Sport University
IOHP: Institute for Occupational Health Promotion
IQIB: Institut für qualifizierende Innovationsforschung und -beratung GmbH
NAO: Network administration organisation
SNA: Social network analysis
WHP: Workplace health promotion
